# Microglia out of place—mapping macrophages across the developing human body

**DOI:** 10.1038/s41423-023-01100-2

**Published:** 2023-11-28

**Authors:** Roman Sankowski, Marco Prinz

**Affiliations:** 1 Institute of Neuropathology, Faculty of Medicine, University of Freiburg, Freiburg, Germany; 2 Signalling Research Centres BIOSS and CIBSS, University of Freiburg, Freiburg, Germany

**Keywords:** Imaging the immune system, Autoimmunity

Macrophages (MΦs) are versatile cells found in every organ. Throughout the body, they work tirelessly to maintain tissues. To this end, they are instructed by their respective niches to express organ-specific genes and proteins, resulting in a variety of cell morphologies and highly specialized functions across different tissues [1]. A recent study by Wang et al. used single-cell RNA sequencing to comprehensively profile human MΦ phenotypes across 19 organs between postconception weeks (PCW) 4 and 26 [2]. This study revealed a surprising diversity of these cells and suggests new avenues of research.

Postnatal MΦs exhibit various morphological and transcriptional phenotypes, ranging from highly branched forms (microglia in the brain and Langerhans cells in the epidermis) to bipolar shapes (perivascular and leptomeningeal MΦs) and even large multinucleated cells (bone osteoclasts) [3, 4]. At all these sites, MΦs constantly survey their surroundings to recognize and remove cell debris, metabolites, and invading pathogens and even regulate blood flow [4, 5]. Reflecting their diverse roles, MΦs express various marker proteins, including general pan-MΦ markers, such as F4/80 and CD11b, and more specialized proteins, such as Mrc1 and Lyve1 in perivascular MΦs and Sall1 and Hexb in microglia in the central nervous system (CNS). Prior to the study by Wang et al., the prenatal landscape of MΦs across different tissues was not fully resolved.

This new study broadens our understanding of human tissue MΦs by identifying microglia-like cells in the fetal epidermis, testicles, and heart. Furthermore, it highlights the presence of proangiogenic MΦs (termed PraMs) across various organs. Questions arise regarding the functions of these cells and their origins.

Human fetal development is marked by extensive organ growth and refinement (Fig. 1A). This process involves cells from different lineages that colocalize, giving rise to parenchymal cells, blood and lymphatic vessels, and nerves, which later intricately interconnect within the adult organ. Wang et al. found that direct contact with neural crest cells induces microglia-like MΦ phenotypes in the human epidermis. Spatial distribution analysis indicates a preferential enrichment of these cells in the skin of the back and the head rather than the limbs or abdomen. This distribution pattern aligns with the pattern of neural crest cell (NCC) migration. Validation experiments confirmed a direct interaction of SOX10^+^ melanocyte precursor cells. Mechanistically, the authors demonstrated that the depletion of microglia-like cells in skin cultures using clodronate liposomes reduced melanocytes, implying the involvement of these microglia-like MΦs in melanocyte differentiation. Later in development, these cells are replaced by Langerhans cells, which are also of fetal origin. In the developing human heart, microglia-like cells accumulate and become the major immune population in the aorta at PCW 26. Remarkably, these cells are absent in adulthood. Future research can delve deeper into the microglia-like cell niche using spatial transcriptome profiling.

**Fig. 1:**
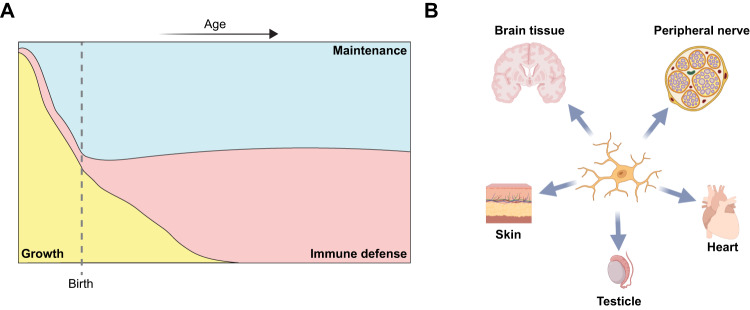
Niche characteristics drive the phenotype of human tissue MΦs. **A** The functional spectrum of tissue-resident MΦs throughout the human lifespan. **B** Structures conveying microglia-like transcriptional states. Panel (**B**) was generated using Biorender.com

The authors acknowledge the uniqueness of their findings, given that the microglia-like phenotype was previously associated exclusively with the brain environment. Recent studies have suggested that peripheral nerve-associated MΦs may share some features with activated microglia [6]. The novelty of the current study lies in establishing NCC-derived cells as a new structure that induces microglia-like transcriptional programs. Other structures include brain tissue, cerebrospinal fluid (CSF) and peripheral nerve fibers (Fig. 1B). However, the specific molecules or processes responsible for this induction remain unclear, warranting further investigation into the underlying molecular mechanisms.

The study, while not pinpointing the mechanisms, maps the journey of progenitor cells as they transform into microglia-like cells. The authors approached this challenge by identifying the commonalities of the sites where microglia-like cells are found. The epidermis and CNS tissues stem from the ectoderm, while the aorta and epididymis originate in the aorta-gonad-mesonephros (AGM) region. The authors propose that precursor cells migrate from the yolk sac to the ectoderm and AGM region, giving rise to microglia or microglia-like cells. Unfortunately, the study does not elucidate how these cells are substituted in the later prenatal stages, which were not analyzed. It would be intriguing to investigate whether these cells are replaced after the removal of survival signals or if they differentiate into Langerhans-like cells when their environment changes. Replacement seems to be plausible, given the distinct position of microglia and Langerhans cells. Furthermore, it remains unclear whether transcriptionally similar microglia-like cells across organs also share typical microglial functional features, such as synaptic pruning, or promote neuronal survival, as in the CNS.

In contrast to the eclectic distribution pattern of microglia-like cells, PraMs were present in the perivascular space across various organs. These cells express generic programs rather than tissue-specific programs, likely due to their localization between organs and blood vessels. Mechanistic experiments showed that CD206^+^CD83^+^ PraMs were more angiogenic than their CD206^+^CD83^low^ counterparts and CD106^-^P2RY12^+^ microglia. The authors suggest that the perivascular niche drives the differentiation of yolk sac-derived MΦ progenitors (YSdMPs) into PraMs.

The ontogeny of MΦs has been extensively explored in mice [7, 8]. Considering the chronological appearance of various MΦ subsets, the authors employed different in silico pseudotime analyses to infer the transitions between them. These analyses suggest that YSdMPs give rise to pre-PraMs, which subsequently generate PraMs. For microglia, which are known to originate from the yolk sac [7], the analysis indicates that they stem from an alpha-fetoprotein (AFP)^high^ population of YSdMPs expressing *AFP* and *TTR*, with primitive head-enriched MΦ progenitors (HeMPs) as an intermediate state. These results aligned well with the observed chronological time. Transcriptionally, the maturation dynamics of microglia and PraMs involve enhanced postnatal expression of major histocompatibility complex (MHC) class II genes. MHC class II genes were already highly expressed in other fetal MΦ subsets, such as Langerhans cells. Transcriptional regulatory factor analysis suggests the involvement of various known and novel transcription factors in different MΦ subsets, such as *SALL1* in microglia and previously unidentified factors, including *IRF5* and *PRDM1*, in other subsets. In sum, the authors provide unprecedented insights into human MΦ ontogeny. Future research will likely focus on the regulatory processes governing the bifurcation of the differentiation trajectories at later developmental and postnatal stages of life.

The birth of an individual exposes the body’s surfaces to the external world. This transition, marking the end of intrauterine immune protection, triggers a reconfiguration of MΦ compartments throughout the body, which previously operated in a protected immune environment. This protection gives the respective tissue MΦs the framework to support organ growth and development while relatively unrestrained by immune duties. Across different organs, MΦs are replaced by cells from definitive hematopoiesis. Only the so-called immune privileged sites, including the brain and eye, continue their developmental trajectories without contact with the outside world. In the brain and eye, microglia remain the main immune self-replenishing cell population throughout life [3, 4].

During development, microglia initially function as phagocytes that upregulate immune effector genes. Throughout development, adulthood and aging, microglia exhibit a range of phenotypes with region- and aging-dependent expression of activation programs [9, 10]. CNS-associated MΦs (CAMs) that reside at CNS borders [11, 12] emerge alongside microglia and evolve as the brain border niche matures postnatally [13]. A notable border region is the choroid plexus, which harbors microglia-like cells at its interface with the cerebrospinal fluid (CSF) [12]. MΦs in the choroid plexus stroma are postnatally replaced by bone marrow-derived MΦs [11], consistent with the concept that the choroid plexus is involved in the immune regulation of the brain. Future research could delve into the maturation of MΦs at the developing brain border regions.

One of the most critical changes occurring at birth is the colonization of body surfaces by microbiota. Throughout life, microbiota continuously train and shape the immune system. Importantly, microbiota-generated metabolites have profound effects on distant sites, including the brain, influencing microglia and CAM phenotypes. Germ-free animals exhibit impaired microglia and brain development and altered responses to brain pathologies [14, 15].

In conclusion, Wang and coauthors, by identifying microglia-like MΦs and PraMs, significantly advance our knowledge of human developmental MΦ states. The study underscores the adaptability of MΦ niches across the body and their crucial role in structural immunity.

## References

[1] Guilliams M, Svedberg FR (2021). Does tissue imprinting restrict macrophage plasticity?. Nat Immunol.

[2] Wang Z, Wu Z, Wang H, Feng R, Wang G, Li M (2023). An immune cell atlas reveals the dynamics of human macrophage specification during prenatal development. Cell.

[3] Prinz M, Masuda T, Wheeler MA, Quintana FJ (2021). Microglia and central nervous system–associated macrophages—from origin to disease modulation. Annu Rev Immunol.

[4] Prinz M, Jung S, Priller J (2019). Microglia Biology: one century of evolving concepts. Cell.

[5] Masuda T, Sankowski R, Staszewski O, Prinz M (2020). Microglia heterogeneity in the single-cell era. Cell Rep.

[6] Ydens E, Amann L, Asselbergh B, Scott CL, Martens L, Sichien D (2020). Profiling peripheral nerve macrophages reveals two macrophage subsets with distinct localization, transcriptome and response to injury. Nat Neurosci.

[7] Kierdorf K, Erny D, Goldmann T, Sander V, Schulz C, Perdiguero EG (2013). Microglia emerge from erythromyeloid precursors via Pu.1- and Irf8-dependent pathways. Nat Neurosci.

[8] Schulz C, Gomez Perdiguero E, Chorro L, Szabo-Rogers H, Cagnard N, Kierdorf K (2012). A lineage of myeloid cells independent of Myb and hematopoietic stem cells. Science.

[9] Masuda T, Sankowski R, Staszewski O, Böttcher C, Amann L, Sagar (2019). Spatial and temporal heterogeneity of mouse and human microglia at single-cell resolution. Nature.

[10] Sankowski R, Böttcher C, Masuda T, Geirsdottir L, Sagar, Sindram E (2019). Mapping microglia states in the human brain through the integration of high-dimensional techniques. Nat Neurosci.

[11] Goldmann T, Wieghofer P, Jordão MJ, Prutek F, Hagemeyer N, Frenzel K (2016). Origin, fate and dynamics of macrophages at central nervous system interfaces. Nat Immunol.

[12] Jordão MJC, Sankowski R, Brendecke SM, Sagar, Locatelli G, Tai YH (2019). Single-cell profiling identifies myeloid cell subsets with distinct fates during neuroinflammation. Science.

[13] Masuda T, Amann L, Monaco G, Sankowski R, Staszewski O, Krueger M (2022). Specification of CNS macrophage subsets occurs postnatally in defined niches. Nature.

[14] Erny D, Hrabě de Angelis AL, Jaitin D, Wieghofer P, Staszewski O, David E (2015). Host microbiota constantly control maturation and function of microglia in the CNS. Nat Neurosci.

[15] Sankowski R, Ahmari J, Mezö C, Hrabě de Angelis AL, Fuchs V, Utermöhlen O (2021). Commensal microbiota divergently affect myeloid subsets in the mammalian central nervous system during homeostasis and disease. EMBO J.

